# Young older adults at risk of not having a good later life and the implications for mental health and wellbeing: evidence from the English Longitudinal Study of Ageing

**DOI:** 10.21203/rs.3.rs-3404200/v1

**Published:** 2023-10-09

**Authors:** Paola Zaninotto, Andrew Steptoe

**Affiliations:** University College London; University College London

**Keywords:** Risk groups, depression, wellbeing, life satisfaction, loneliness

## Abstract

We identified risk categories of not having a good later life among young older people and reported the consequences that being in these groups have on mental health and wellbeing.

3,511 participants aged 50 to 69 from the English Longitudinal Study of Ageing (ELSA) provided data on 10 domains of a good later life. The domains were then entered into a model to identify risk groups using Latent Class Analysis. Regression models were used to assess the association between identified risk groups and depression, life satisfaction, loneliness and wellbeing. We found that 20% of individuals were in the “high” risk group for not having a good later life. These people were more likely to report depression, loneliness, lower life satisfaction and lower wellbeing than those in the low risk group. Being at risk of not having a good later life has important consequences for mental health and wellbeing. Appropriate support and services should be in place to ensure that everyone is able to live longer in good mental health.

## Introduction

The world’s population is ageing at a rapid pace, as a result, the number of people approaching later life is also rising significantly. In England, 3 in 1 adults are aged 50 to 69 (13.5 million), and this number is expected to increase by 6% in 2030 (14.3 million) (Statistics, 2019). This represents a substantial demographic group whose later life will be most likely influenced by current personal health, work, social and financial circumstances, which in turn will affect the rest of society and the future planning of public services and policies.

Later life can be seen as a period of life in which one is free to explore personal fulfilment, self-realisation and leisure, since there is more autonomy from structured social roles (e.g. employment, parent of dependent children) (Bowling, 2005; Laslett, 1996). However, the experience of later life can be very diverse. Millions of people worldwide are missing-out on a long, healthy and fulfilling later life (Mokdad et al., 2016), with chronic ill-health and lack of economic, psychological and social resources frequently contributing to a lack of flourishing (Pavalko & Caputo, 2013; Schotanus-Dijkstra et al., 2016). To this extent, it is important to better understand aspects of people’s lives in the years approaching later life, which often determine whether or not individuals will be able to enjoy a good later life. These aspects include social connections, financial circumstances, work, housing, health and healthy lifestyles to maintain health and wellbeing (Bosnes et al., 2019; Depp & Jeste, 2006). The years from midlife to early old age are an important period of life, in which interventions, adjustments and planning can take place to ensure that individuals enjoy fulfilling later lives (Myint et al., 2011). Nevertheless, this age group has been the focus of less research than many other sectors of the population, and little is known on their risk of missing out on a good later life based on current circumstances (Lafortune et al., 2016).

## Conceptual Framework

This study seeks to identify groups of individuals aged 50–69 at risk of not having a good later life using as conceptual framework the strategy developed by the Centre for Ageing Better (Ageing-Better-Transforming-Later-Lives.pdf) for transforming later life. This is the first time that this framework is applied to research. The Centre for Ageing Better is a UK-based charitable organization that focuses on promoting positive and fulfilling experiences of aging for individuals in later life. Established in 2015, the Centre for Ageing Better operates as an independent entity, working in collaboration with a range of partners, including government, non-profit organizations, academia, and businesses. The organization’s core mission is to bring about positive change in policies, practices, and attitudes that affect older adults, aiming to create a society where individuals can enjoy a good later life. The Centre for Ageing Better conducts research, generates evidence-based insights, and develops practical solutions to address the challenges and opportunities associated with aging. The conceptual framework has identified several key areas relevant to the enjoyment of a good later life: Good Health, Healthy Ageing, Social Connections, Connected Communities, Meaning and Purpose, Financial Security, Safe and Accessible Housing, Inclusive Planning and Design, Affordability, Fulfilling Work and Work and Health. Good Health refers to the risk of poor health (including health issues that impair the ability to work), delaying the onset and progression of chronic conditions; Healthy Ageing refers to being physically active, not engaging in unhealthy lifestyles such as smoking and drinking, and maintaining good cognitive health. Social Connections and Connected Communities refer to connections with other people, to a sense of belonging to a community, group or society, to having close connections with family and friends as well as to a wider sense of Meaning and Purpose. Financial Security involves ensuring income is adequate for daily life, with sufficient savings to cope with future emergencies; Affordability refers to whether or not people can make ends meet. Safe and Accessible Housing refers to the ability of individuals to live in safe, accessible and adaptable homes, which allow then to remain independent and active for longer. Inclusive Planning and Design refers to the elimination of barriers to suitable transport when needed. Lastly, factors that make Fulfilling Work include being in a good quality work characterised by control and satisfaction, and a balance between efforts and reward; Work and Health refers to the health and wellbeing of people in workforce.

By addressing these key areas, the framework developed by the Centre for Ageing Better aims to transform later life experiences, promoting positive aging, and creating an environment where older adults can thrive and enjoy a fulfilling and meaningful later life. Using the latest data from a large nationally representative sample of older adults in England, participants of the English Longitudinal Study of Ageing (ELSA)(Steptoe et al., 2013), this study has several objectives. First, we operationalise the domains of a good later life using the Centre for Ageing Better conceptual framework. Second, using these domains, we group individuals into risk categories, to understand whether the conceptual framework can be successfully used for this purpose in research. Lastly, we explore the consequences of being at risk of not having a good later life by reporting the associations between risk groups and mental health and wellbeing.

## Methods

### Data source

For the purpose of this study we used the latest assessment (wave 9 2018/2019) of the English Longitudinal Study of Ageing (ELSA)(Steptoe et al., 2013; Zaninotto & Steptoe, 2019). Started in 2002, ELSA is an ongoing prospective cohort study of nationally representative older adults aged 50 years and over, living in private households in England. The aim of ELSA is to further our understanding of the ageing process by exploring multiple objective and subjective facets of people’s lives (https://www.elsa-project.ac.uk/). Data are collected at both household and individuals level. A computer-assisted personal interview is the primary means of data collection, supplemented with a self-completed questionnaire. In addition, the dataset is enriched periodically by clinical data from nurse visits. The total sample interviewed in Wave 9 (2018/2019) consisted of 8,736 individuals aged 50 to 100, however 6,445 participants aged 50 to 100 provided data on the main interview and self-completion questionnaire (see Sample size derivation chart in Supplementary figure 1). Of these we selected 3,511 individuals aged 50 to 69 interviewed, which formed our analytical sample. Participants gave their consent to participate in the study, ethical approval was received from an Ethics Committee on 10th May 2018 (17/SC/0588).

### Measures

#### Domains of a good later life

Based on information collected during the ELSA interviewer we operationalised the following 10 domains of a good later life (see page 5 of Ageing-Better-Transforming-Later-Lives.pdf): Good Health, Healthy Ageing, Social Connections, Inclusive Planning and Design, Meaning and Purpose, Affordability, Financial Security, Safe and Accessible Housing, Fulfilling Work and Work and Health. A total of 29 characteristics were identified and structured theoretically, with help of the Centre for Ageing Better, as being markers of the ten domains of a good later life and these are described below. The domains are mutually exclusive and they evaluate different areas of later life. *Good Health* was defined as the absence of: a major chronic condition (angina, myocardial infarction, other heart problems, stroke, diabetes, arthritis, high blood pressure, dementia/Alzheimer’s/Parkinson’s, multiple sclerosis), a limiting longstanding illness, poor self-rated health, hearing impairment and/or vision impairment. The following characteristics were used to capture *Healthy Ageing* focusses on behavioural aspects of health, such as engaging in any of the following behavioural risk factors: current smoking, physically inactive and daily alcohol consumption. *Fulfilling Work* included information on excessive job demands, effort/reward imbalance and lack of control at work. *Work and Health* included information on whether the respondent undertook substantial heavy manual work and/or illness and disability limited the amount of work done. *Meaning and Purpose* was measured using information on whether respondents often looked forward to each day and whether they stated that their life has meaning. *Social Connections* were captured using respondents’ rating on relationships with the partner, children, close relatives and friends, on membership to clubs society and organisations and on engaging in voluntary work. *Inclusive planning* and design was defined as having or not access to suitable transport when needed. *Safe and Accessible Housing* included information regarding problems with the accommodation such as excess noise, lack of space, condensation/dump, cold, pollution, water leaks, pests. *Financial Security* included information on net wealth, weekly income and whether or not respondents had enough money to meet future needs. *Affordability* was defined as having or not enough money to buy food and for immediate needs. Additional information regarding each item is provided in Supplementary materials (Supplementary Table 1).

#### Outcome variables

We focused on the following outcomes: depression, wellbeing and life satisfaction. Depressive symptoms were ascertained using the 8-item Centre for Epidemiological Studies Depression (CESD-8) scale, which measures eight different symptoms of depression (e.g. “felt depressed”, “everything I did was an effort”, “sleep was restless”). This scale has previously been validated against gold-standard psychiatric interviews with good sensitivity and specificity(Radloff, 1977). A dichotomous (yes/no) response was used for each item, resulting in a total CESD-8 score ranging between zero (no symptoms) and eight (all eight symptoms). We then created a binary variable using a cut-off point of four or more symptoms to identify likely cases of clinical depression, which is equivalent to the conventional threshold of 16 or higher on the full 20-item CESD scale (Steffick, 2000). A wellbeing score was derived using four questions of the ONS Wellbeing scale (ONS, 2018): “Overall how happy did you feel yesterday?”, “Overall, how anxious did you feel yesterday?”, “Overall, to what extent do you feel the things you do in your life are worthwhile?” and “Overall, how satisfied are you with your life nowadays?”. Respondents ranked their responses on a scale from 0 (not at all) to 10 (very). Scores were summed to compute an overall score (ranging from 0 to 40), with higher scores indicating better wellbeing. Loneliness was measured with a single item “How often do you feel lonely” of the Loneliness scale (Hughes et al., 2004) which was rated on a 3-point scale (1 = “hardly ever/never”; 2 = “some of the time”; 3 = “often”). A binary variable was derived to define people experiencing loneliness (some of the time or often). We measured life satisfaction with the Satisfaction with Life Scale (SWLS) (Diener et al., 1985). This consists of five statements about overall satisfaction with life. Possible responses to these statements ranged from 6 (strongly agree) to 0 (strongly disagree). The life satisfaction summary score ranged from 0 to 30, with higher values reflecting greater satisfaction with life.

#### Other variables

The following additional variables were used: age, sex, ethnicity (white vs non-white), retirement status (completely retired, in paid work, other - looking after home and family, currently out of work, permanently unable to work), living or not with a partner, average weekly income and average annual wealth (defined as financial, plus physical -such as business wealth, land or jewellery-plus housing wealth, minus debts).

### Statistical analysis

Our analytical sample was comprised of 3,511 individuals aged 50 to 69 with valid data on all variables of interest. All statistical analyses accounted for the complex survey design and used survey weights to account for non-response. The 10 domains of a good later life were created from the 29 characteristics described in Supplementary materials (Supplementary Table 1). Each of the 10 items was coded as 0”not at risk” and 1”at risk” to identify individuals that currently were at risk of not reporting a good later life on that item. Those not employed were considered as not missing out in the items of Fulfilling work and Work and Health. The 10 items were then entered into a model to identify risk groups of good later life using Latent Class Analysis (Hagenaars & McCutcheon, 2002). Latent Class Analysis is a statistical method for identifying class membership among subjects using several characteristics. The probability of an individual belonging to each class was then used to assign individuals to the most appropriate risk group (the one they had the greatest probability of belonging to). Initially we entered all 29 items into the model, however, there was a suggestion of sparse cells in the cross-classification tables, contributing to less stable models. Therefore, we presented as the final model the one that used the 10 domains described above. We tested models with one, two, three and four classes and based on the model fit, meaning of the classes and number of observations in each class we chose the model with three classes (Garrett & Zeger, 2000), supplementary Table 2. We then considered the domains’ posterior probabilities (≥0.4) (supplementary Table 3) that loaded into each class (group) to name them as: Low risk; Medium risk and High risk. Note that the probabilities in supplementary Table 3 refer to negative aspects of each domain. We found that only domain Healthy ageing loaded on the Low risk group; whereas Good Health, Healthy Ageing, Social Connections, Work and Health and Fulfilling Work loaded on the Medium risk group. Lastly, several domains loaded on the High risk group: Good Health, Healthy Ageing, Social Connections, Meaning and Purpose, financial security, Safe and Accessible Housing and Work and Health. The risk groups were saved into a variable which was then used in regression analyses as an exposure to explore the effects of being in each good later life risk group on outcomes: depression, loneliness, wellbeing and life satisfaction. Regression models were adjusted for age, sex, ethnicity, working status and living or not with a partner.

## Results

### Sample characteristics

Sample characteristics are described in Table 1. The sample was predominantly of white ethnicity, there were slightly more women than men (52%) and nearly one in five people lived alone. The average weekly income for the poorest group was £143 compared to £1036 for the richest. We find huge disparities in net wealth between the richest and poorest. It would take the average combined net wealth of 200 people in the poorest wealth group (bottom 20% of wealth distribution) to equal the average value of a single person in the richest group (top 20% of the wealth distribution). Around a third of people had retired completely and more than half were still in paid work. About 20% of respondents reported being depressed, and 32% reported being lonely. The average wellbeing score was 25 (out of a maximum of 40) and the average life satisfaction score was 21 (out of a maximum of 30).

### Good later life domains

In Table 2 we present the prevalence of people missing out on each domain of a good later life. Most commonly people in this sample were at risk of not reporting Healthy Ageing (49%), Good Health (39%), Social Connections (38%), Financial Security (39%), Fulfilling Work (37%) and Safe and Accessible Housing (35%). One in five reported missing out on Work and Health and one in ten on Meaning and Purpose. A small proportion of people were at risk in the domains of Affordability (8%) and Inclusive Planning and Design (7%). There were very few gender differences in the Good Later life domain: women compared to men were at higher risk due to lack of inclusive planning and financial security. Men by contrast, were at higher than women due to lack of Work and Health and Healthy Ageing (data not shown). In Supplementary Table 1 we can see the % of the characteristics in each domain. Having a major long term condition was the most prevalent in Good Health (66%), poor memory (23%) in Healthy Ageing, not volunteering and poor relationships with friends in Social Connections (44% and 38% respectively), excessive work demands (52%) in Fulfilling Work, whereas characteristics in the other domains were more equally present.

### Risk groups for the likelihood of having a good later life

**Figure 1:**
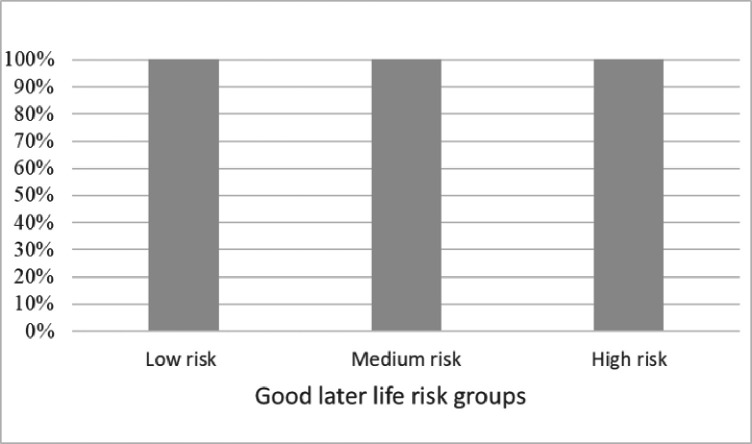
Risk groups for the likelihood of not having a good later life (N=3,511), ELSA 2018 Note: Low: at risk on Healthy Ageing. Medium: at risk on Good Health, Healthy Ageing, Social Connections, Work and Health and Fulfilling Work High: at risk on Good Health, Healthy Ageing, Social Connections, Meaning and Purpose, Financial Security, Safe and Accessible Housing and Work and Health.

Based on the domains of a good later life reported in Table 2, we grouped people into “low”, “medium” and “high” risk groups using latent class analysis. Just over half of the sample was classified at “low” risk for not having a good later life, whereas 23% of people were in the “medium” risk group and 20% in the “high” risk group (Fig. 1). No gender differences were found in these patters.

### Consequences of missing out on a good later life on mental health and well

**Figure 2:**
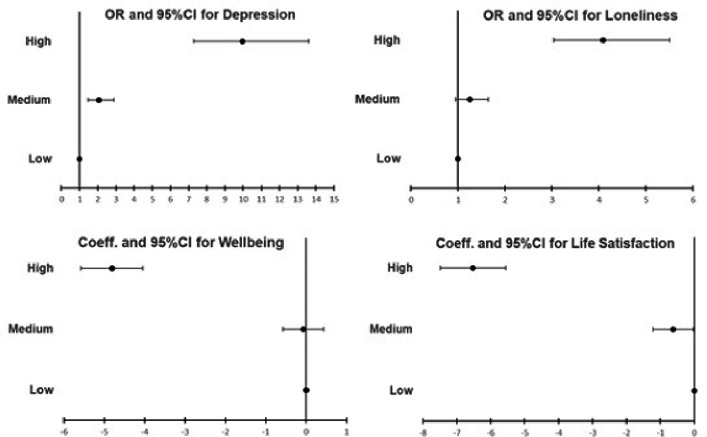
Odds ratios and regression coefficients (95%CI) for the associations between risk groups for the likelihood of not having a good later life and Depression, Loneliness, Wellbeing and Life satisfaction (N=3,511), ELSA 2018 Estimates account for the complex survey design and non-response weights. Estimates adjusted for age, sex, ethnicity working status and living or not with a partner

In Fig. 2 we report the adjusted odds ratios (for binary outcomes) and regression coefficients (for continuous outcomes) and corresponding 95% Confidence Intervals, for the associations between the good later life risk groups and several outcomes: depression, loneliness, wellbeing and life satisfaction. Those in the medium risk group compared to those at low risk, had greater odds of experiencing depression (OR:2.0, 95%CI: 1.5; 2.9) and reported lower life satisfaction scores (coeff:−0.63 95%CI: −1.2; −0.02). Participants in the high-risk group for not having a good later life were more likely to experience all outcomes than the low risk group, with a 4-fold increase in odds of experiencing loneliness (OR:4.1, 95%CI: 3.0; 5.5) and 10-fold increase in odds of depression (OR:10, 95%CI:7.3; 13.6).

## Discussion

In this study, our first objective was to employ a unique framework to operationalize ten domains that encompass a good later life among a large nationally representative sample of individuals aged 50 to 69 residing in England. With the anticipation that a significant proportion of young older adults may not be experiencing these domains fully, we sought to investigate their prevalence. The findings revealed that among the participants in this sample, they most frequently missed on aspects such as Healthy Aging, Good Health, Social Connections, Financial Security, Fulfilling Work, and Safe and Accessible Housing, which are important and will have consequences on how these young older adults will approach older age, in terms of progression and impact of disease (Grant et al., 2009), remaining independent for longer (Shankar et al., 2017), and survival (Zaninotto, Batty, et al., 2020). The high proportion of participants at risk for not reporting Good Health (39%) is a reflection of the fact that many of the chronic long-term conditions of older age first emerge in this age group (MacNee et al., 2014). In part, this is an indication of the substantial numbers who are at risk also of not reporting Healthy Ageing (49%) on account of physical inactivity and alcohol consumption. Adopting healthy lifestyle in young old age has consequences on health in later life, as it has been shown that improving physical activity and reducing alcohol consumption may promote survival (Bowling, 2004), longevity (Zaninotto, Head, et al., 2020) and decrease the risk of serious health adverse outcomes (Shankar et al., 2010), including frailty (Gil-Salcedo et al., 2020).

It is striking that more than one-third of respondents were missing out in terms of Social Connections. The problem of social isolation at older ages is well recognised (Holt-Lunstad et al., 2017; Holt-Lunstad & Steptoe, 2021), results from the National Health and Aging Trends Study showed that 28% of people aged 65–69 reported social isolation (Cudjoe et al., 2020). Social isolation in turn impacts on future health outcomes. A longitudinal analysis of the National Health and Aging Trends Study restricted to people aged 65 + showed that a 1-unit increase in the Social Isolation Index resulted in an average decrease of 0.27 units in the Short Physical Performance Battery (Cruz et al., 2021). Whereas, evidence from ELSA (age range 50 to 100) showed that social isolation is negatively related to cognitive function (Shankar et al., 2017).

The high percentages of people at risk on Safe and Accessible Housing (35%) is an inditement of the quality of the housing stock in England with many people suffering from crowded, noisy, and poorly heated and insulated accommodation. Recent results from the English Housing Survey (Rottier, 2020) showed that in 2019 4.1 millions of homes in England failed to meet the Decent Home Standard, with the majority being in the private rented sector. Poor housing conditions have been associated with poor mental health (Evans et al., 2003), physical functioning limitations (Perez-Hernandez et al., 2018) and respiratory diseases (Webb et al., 2013) at older ages. People at younger old age might be particularly affected and at higher risk of poor health outcomes, as data from Office for National Statistics has shown that increases from the private rental sector are most pronounced in mid-life (ONS, 2020).

It is therefore evident that being at risk in any of these domains will certainly have important consequences for older people, however, some might argue that being at risk on one or a few of these domains might not necessarily translate into less enjoyable older age. To understand whether this might be the case, and to explore whether the conceptual framework can be successfully used for this purpose in research, the second aim of this study was to group individuals into risk categories of not having a good later life based on their risk status the items described above. We found three risk categories of individuals, confirming that the conceptual framework can be successfully applied to research. One in five people were in the high risk group, consisting on those at risk of Good Health, Healthy Ageing, Social Connections, Meaning and Purpose, Financial Security, Safe and Accessible Housing and Work and Health. We showed how the interaction of these domains contributed to risks of poor mental health and wellbeing in later life.

Out last aim was to explore the consequences of missing out on a good later life by reporting the associations between risk groups and outcomes such as mental health, loneliness, wellbeing and a life satisfaction. We showed that people in the high risk group, compared to those in the low risk group (i.e. at risk on Healthy Ageing only), reported higher levels of depression, loneliness, as well as lower wellbeing and life satisfaction.

The framework acknowledges the idea that older adults can experience a sense of enjoyment and fulfilment in their lives despite not being in good health or in the presence of chronic disease or cognitive impairment. It recognizes that a good later life is a multidimensional construct that goes beyond physical health and cognitive functioning. By considering diverse domains, the framework provides a more holistic understanding of older adults’ lives. It recognizes that even with the challenges posed by chronic disease or cognitive impairment, individuals can still find meaning, engage in meaningful relationships, pursue personal interests, and have a sense of purpose and satisfaction in their lives, in line with other studies on wellbeing in later life (Blane et al., 2004; Lee et al., 2021; Wiggins et al., 2004). In doing so, the framework challenges the notion that a good later life is solely determined by health status. It emphasizes the importance of creating supportive environments, fostering social connections, promoting connected communities, fulfilling work and enabling individuals to maintain their autonomy and agency. This perspective allows for a more comprehensive assessment of older adults’ lives and opens up opportunities for interventions and policies that can enhance their quality of life, despite the presence of chronic disease or cognitive impairment. Direct comparisons with other conceptual frameworks was not possible as to our knowledge there has not been an attempt to conceptualise a good later life using domains such as housing, financial security, etc alongside health and wellbeing.

Overall, the policy implications of these results emphasize the need for comprehensive approaches that address multiple domains of a good later life. Interventions should focus on promoting healthy behaviours, strengthening social connections, ensuring financial security, facilitating fulfilling work opportunities, and improving housing conditions. Support aimed at improving working conditions, affordable and suitable housing options, and improve accessibility and safety standards, will allow young older adults to maintain their independence for longer, contribute to improved mental health outcomes, enhanced quality of life, as they approach older age.

This study has several strengths. First it involved a holistic analysis of the experience of ageing, involving a wide range of aspects from health through social and economic issues to living environments. Such an analysis is only possible using a multidisciplinary study like ELSA which gathers information across a broad spectrum of topics relevant to later life. Second it employed a large nationally representative sample of older adults living in England. Furthermore, the Centre for Ageing Better’s framework presented here offers a unique perspective by encompassing domains that are often overlooked in the existing wellbeing literature, such as safe housing. This novel approach takes into account crucial structural social factors that significantly contribute to a comprehensive understanding of wellbeing in older age. By considering these factors within the broader conceptualization of wellbeing, the framework holds significant potential for meaningful applications in policymaking. Lastly it used a state-of-the-art methodological techniques to classify individuals into risk groups (latent class analysis). A possible limitation of our work is that the categories of good later life might differ in studies employing different domains of a good later life or different characteristics. We used the Centre for Ageing Better’s conceptual framework, but future studies might consider a more restricted or wider set of characteristics to conceptualise good later life. Finally, our analyses are restricted to those aged 50 to 69, different results might be obtained in a population with a different age composition, i.e. people over the age of 70 might be classified in the high risk group.

To conclude our study showed that a large number of young older adults are high risk group for not having a good later life and that this in turn has important consequences in terms of mental health, wellbeing, and life satisfaction. Considering that these adults are soon approaching later life interventions should be in place to change their trajectory through appropriate support and services to make sure that everyone is able to live longer in good conditions.

## Figures and Tables

**Table 1:** Sample characteristics (N = 3,511), ELSA 2018

**Female % (N)**	**57 (2003)**
**Mean age in years (S.D)**	60 (6.1) range 50 to 69
**Non-White Ethnicity % (N)**	8 (282)
**Lives alone % (N)**	19 (659)
**Retirement status**	
Completely retired **% (N)**	29 (1009)
In paid work **% (N)**	57 (2008)
Other^a^ **% (N)**	14 (494)
**Quintiles of wealth**	**Mean annual Wealth (£) (S.D)**
Poorest	6582 (17564) range - 39650 to 66500
2	144856 (42645) range 67300 to 218600
3	302671 (49094) range 218800 to 389100
4	514052 (78255) range 389400 to 675000
Richest	1353392 (1021178) range 676500 to 9695200
**Quintiles of income**	**Mean weekly income (£)**
Poorest	143 (73.1) range 0 to 285
2	281 (50) range 265 to 376
3	387 (51) range 336 to 461
4	527 (52) range 451 to 641
Richest	1036 (980) range 808 to 8964
**Depression % (N)**	20 (688)
**Loneliness % (N)**	32 (1116)
**Mean score of Wellbeing (S.D)**	25 (7.1) range 2 to 50
**Mean score of Life satisfaction (S.D)**	21 (6.3) range 0 to 30

Notes: Estimates account for the complex survey design and non-response weights.

a. Other: looking after home and family, currently out of work, permanently unable to work

**Table 2:** Prevalence of people at risk on each domain of a good later life N = 3,511, ELSA 2018

**Good later life domains**	
**Good Health**	
Not at risk	61%
At risk	39%
**Healthy Ageing**	
Not at risk	51%
At risk	49%
**Social Connections**	
Not at risk	62%
At risk	38%
**Inclusive Planning and Design**	
Not at risk	93%
At risk	7%
**Meaning and Purpose**	
Not at risk	88%
At risk	12%
**Affordability**	
Not at risk	92%
At risk	8%
**Financial Security**	
Not at risk	61%
At risk	39%
**Safe and Accessible Housing**	
Not at risk	65%
At risk	35%
**Work & Health**	
Not at risk	79%
At risk	21%
**Fulfilling Work**	
Not at risk	63%
At risk	37%

Estimates account for the complex survey design and non-response weights.

## Data Availability

This study uses data from the English Longitudinal Study of Ageing, which are freely available upon registration through the UK Data Service. NatCen Social Research, University College London, Institute for Fiscal Studies. (2023). *English Longitudinal Study of Ageing*. [data series]. *7th Release*. UK Data Service. SN: 200011, DOI: http://doi.org/10.5255/UKDA-Series-200011
